# Detecting and Predicting Emerging Disease in Poultry With the Implementation of New Technologies and Big Data: A Focus on Avian Influenza Virus

**DOI:** 10.3389/fvets.2018.00263

**Published:** 2018-10-30

**Authors:** Jake Astill, Rozita A. Dara, Evan D. G. Fraser, Shayan Sharif

**Affiliations:** ^1^Department of Pathobiology, University of Guelph, Guelph, ON, Canada; ^2^School of Computer Science, University of Guelph, Guelph, ON, Canada; ^3^Arrell Food Institute and Department of Geography, Environment and Geomatics, University of Guelph, Guelph, ON, Canada

**Keywords:** influenza virus, poultry, rapid diagnosis, big data, biosensor, infectious disease

## Abstract

Future demands for food will place agricultural systems under pressure to increase production. Poultry is accepted as a good source of protein and the poultry industry will be forced to intensify production in many countries, leading to greater numbers of farms that house birds at elevated densities. Increasing farmed poultry can facilitate enhanced transmission of infectious pathogens among birds, such as avian influenza virus among others, which have the potential to induce widespread mortality in poultry and cause considerable economic losses. Additionally, the capability of some emerging poultry pathogens to cause zoonotic human infection will be increased as greater numbers of poultry operations could increase human contact with poultry pathogens. In order to combat the increased risk of spread of infectious disease in poultry due to intensified systems of production, rapid detection and diagnosis is paramount. In this review, multiple technologies that can facilitate accurate and rapid detection and diagnosis of poultry diseases are highlighted from the literature, with a focus on technologies developed specifically for avian influenza virus diagnosis. Rapid detection and diagnostic technologies allow for responses to be made sooner when disease is detected, decreasing further bird transmission and associated costs. Additionally, systems of rapid disease detection produce data that can be utilized in decision support systems that can predict when and where disease is likely to emerge in poultry. Other sources of data can be included in predictive models, and in this review two highly relevant sources, internet based-data and environmental data, are discussed. Additionally, big data and big data analytics, which will be required in order to integrate voluminous and variable data into predictive models that function in near real-time are also highlighted. Implementing new technologies in the commercial setting will be faced with many challenges, as will designing and operating predictive models for poultry disease emergence. The associated challenges are summarized in this review. Intensified systems of poultry production will require new technologies for detection and diagnosis of infectious disease. This review sets out to summarize them, while providing advantages and limitations of different types of technologies being researched.

## Introduction

Global population growth, along with rising affluence in Asia, are driving up not only our total demand for food, but also the amount of protein required to feed all of humanity (1, 2). More specifically, the world's population is expected to grow to over 9 billion people by 2050, and demand for poultry, which represents a relatively healthy and efficient source of protein, is likely to be double from what it was in 2005. At the same time, it is expected that the world will consume 40% more chicken eggs (3). Reaching these levels of production requires intensification of poultry operations, and this will translate into larger farms with more poultry houses and birds. A concern is raised in the literature that intensive systems of livestock production may be more vulnerable to outbreaks of disease in both farmed animals and in human populations (4, 5). Despite such worries, technological advancements that make it possible for farmers to manage the health status of more birds with less resources are a current source of research and development. Such technologies include biosensors, wearable technologies, and non-invasive approaches of poultry disease surveillance. Additionally, these intensive systems will be able to utilize data captured from various sensors and devices, allowing farmers to better monitor and control both the birds and their environment as a result of better decision making when concerning management. The ability to analyze data in real-time may ameliorate threats of emerging infectious diseases as these tools can allow farmers to stay informed about the health and welfare status of large numbers of poultry. In light of both the rising threat of infectious diseases from larger poultry operations, and the potential for technology to improve diagnostics, surveillance, and early detection, the purpose of this review paper is to explore the current scientific understanding of how technology can be utilized to decrease the threat of infectious diseases within the poultry industry.

When considering infectious disease, it is essential to determine precisely when and where infection is occurring on poultry farms so action can be taken sooner to prevent additional infection and losses. This will not only help improve productivity but is also important from a human safety point of concern, as some poultry pathogens such as avian influenza virus (AIV), pose a significant risk of human infection and possible pandemics in human populations (6). Additionally, other poultry pathogens such as *Escherichia coli, Salmonella* and *Campylobacter jejuni* are capable of inducing human disease (7–9). Incidence of emerging disease in livestock animals and humans is increasing, and a primary reason for this is increased contact between wild animal species, livestock, and humans (10). Additionally, poultry production will be forced to increase at a time where global environmental changes are occurring, and as humans continue to expand the boundaries of cities, living areas, and agricultural land. All of these factors increase the risk of emergence of new infectious diseases into the human population. The emergence of highly pathogenic H7N9 AIV in China is an example of the problems associated with intensification of poultry production, which increases the density of poultry populations leading to more opportunities for transmission between birds and potentially humans (11). Optimal strategies that will help reduce the threat of emerging disease in poultry include the ability to rapidly detect and diagnose bird disease as quickly as possible, and the ability to predict outbreaks. Predicting infectious diseases and initiating rapid responses has great value for both the safety of human populations and for the poultry industry. For example, in 2014 official detection of a highly pathogenic AIV (HPAIV) H5N2 in British Columbia turkey and chicken farms led to their quarantine on December 2 by the Canadian Food Inspection Agency (12). However, initial signs of infection were noted on these farms on November 26 and 28, meaning up to 5 days passed between the onset of disease and actions being taken to reduce spread. Ultimately, HPAIV infection spread to multiple other farms in Canada and the USA over the following weeks and months, and this led to the culling of more than 48 million birds (13). Situations in the future can be handled better by decreasing the time that passes between initial poultry infection and an end diagnosis. Sensor and surveillance technologies can allow for much faster detection of bird disease in addition to providing a rapid on-site diagnosis. This will allow for counteractions to be taken sooner, for example by quarantining poultry houses or farms, thereby decreasing the chance that infection can spread to other groups of poultry.

The ability to contain infectious disease on poultry farms could benefit immensely from systems that first can rapidly detect unhealthy or sick birds, and secondly devices that can accurately and rapidly determine the causative agent that led to disease. Devices that make this a reality are a current source of research, and comprise a variety of different technologies, including multiple types of biosensors and rapid-assays, real-time poultry analysis tools that utilize audio or visual components to assess poultry health, and wearable sensors that additionally can transmit and analyze data about the health of poultry. The many devices, or systems, that could be used to rapidly detect poultry infection and disease vary in strategy, each having their own advantages and disadvantages. In this review, some of these technologies are described and are grouped into three categories: biosensors, wearable poultry sensors, and non-invasive non-contact analysis mechanisms. Additionally, the advantages and drawbacks of each category of devices will be discussed concerning their role in detecting and predicting emerging disease in poultry.

Predicting infectious disease in poultry is becoming possible as new technologies are increasing the availability of data that can be utilized in predictive models. The data generated from rapid detection and diagnostic technologies will be a very important source, however, other data sources can also be used to predict the likelihood of emerging disease in poultry. Drivers of poultry disease must be taken into account for producing predictive models, and these include a wide range of environmental and geographical inputs such as climate, weather, reservoir host species distribution, and livestock density. Additionally, web-based search query and social media data, which have both been suggested to be effective data sources for predicting human influenza virus incidence (14), could also be utilized to predict and track outbreaks and emerging diseases in poultry. Data collected from rapid detection technologies in addition to the above-mentioned data sources can also be factored into decision support systems that can help to determine risk in outbreak situations, leading producers to make better decisions concerning management. In this review, select sources of data that are relevant to emerging disease in poultry will also be highlighted and discussed. Additionally, big data and big data analytics will be highlighted in the context of designing predictive models for disease emergence in poultry, in addition to data-driven decision support systems that enhance decision making pertaining to management practices when faced with the threat of emerging disease.

## Part I

### New technologies that enable rapid detection and diagnosis of infectious disease in poultry

Technologies that are being developed for detecting infection or disease in poultry are widespread and are applicable to multiple infectious agents or physiological conditions (15, 16). The focus of this section is technologies concerned with rapid diagnosis of poultry infection, focused on AIV, as this is an important emerging pathogen in the poultry industry and is the focus of many research efforts. Due to the production of novel influenza viruses through constant mutation and gene assortment, combined with their high prevalence in wild bird reservoirs, AIV constantly threatens to infect farmed poultry and is capable of causing significant economic losses in the industry (17). Traditional diagnosis of AIV in poultry consists of laboratory intensive processes including culture techniques followed by real-time polymerase chain reaction (RT-PCR), in addition to enzyme linked immunosorbent assays (ELISA) (16). Albeit sensitive, these laboratory focused methods can take up to days to reach a diagnosis of AIV infection and they require manual labor from experienced personnel (15). Additionally, some areas where poultry is farmed do not have local laboratories that can facilitate AIV diagnosis, requiring extensive transport of samples and further delay of a diagnosis (17). Rapid detection kits for influenza virus are currently available. These kits can visually signal the presence of influenza virus through detection of viral proteins, however, they often exhibit low sensitivity (18, 19). Rapid and sensitive diagnosis of AIV infection in poultry could be crucial for curbing transmission of the virus during poultry outbreak situations. Multiple technologies are being developed that can help facilitate this. Table 1 provides a summary of the advantages and limitations of different types of technologies for rapid detection or diagnosis of infectious diseases in poultry.

**Table 1:** Summary of potential technologies that facilitate rapid detection or diagnosis of disease in poultry.

**Detection/diagnosis technology**		**advantages**	**limitations**
Biosensors		Specific detection of infectious pathogen causing disease High sensitivity Decrease need for lab diagnosis	Requires manual use Devices often limited to detection of single microorganism
Wearable sensors		Constant surveillance of birds Can detect disease in real-time Can detect different infectious diseases Internet connection facilitates immediate alarm to producer	Does not provide specific diagnosis of infectious pathogen causing disease Only feasible to use on a representative sample of a flock Issues with battery life and sensor weight
Non-contact methods	Image analysisVocalization analysis	Constant surveillance Can detect patterns of disease in real-time Does not require handling of birds Has additional uses beyond detection of infectious disease (weight and behavior analysis)	Does not provide specific diagnosis of infectious pathogen causing disease Background sounds/visuals in poultry houses can disrupt detection
	Robot vehicle surveillance	Autonomously driven and monitor birds constantly Able to detect diseased or dead birds Increase chicken activity Increase barn sanitation	Does not provide specific diagnosis of infectious pathogen causing disease Potential issues with internet connectivity

#### Technology to identify poultry disease: biosensors for influenza virus

When compared to standard laboratory methods, a potential drawback to rapid diagnostic systems is a lack of certainty when forming a diagnosis. For example, most rapid diagnostic kits for human influenza infection have lower levels of sensitivity and they can only detect influenza virus at the species level, detecting whether a sample is positive for *influenza A virus* or *influenza B virus* (19). Diagnosing AIV infection in poultry beyond the virus species level is important, as differentiating between high and low pathogenic viruses is crucial. Nevertheless, biosensors capable of very specific detection are being developed, and offer the potential to detect and differentiate between specific subtypes of AIV.

Biosensors can be described as devices that can convert the presence of a biological element into a signal that is recognizable to the user (20). Functionally, a biosensor can be split into multiple segments including the bioreceptor, which is responsible for recognition of a biological analyte, a transducer, which converts detection of the biological analyte into a signal, electronics, which convert a signal into a displayable result often requiring amplification or conversion of the signal, and a display, which supplies the user with a readable output (21). Recognition by the bioreceptor is facilitated by binding of a recognition element that is usually fixed, and can include antibodies, proteins, nucleic acids, enzymes, aptamers, cells, or other molecules (15). Detection of influenza viruses can be done using recognition elements such as glycans, which can be divided into α-2,6 or α-2,3 sialic acids allowing for partial differentiation between human and avian viruses, antibodies, and aptamers, both of which are capable of detecting specific influenza virus subtypes (22). Additionally, multiple different mechanisms of signal transduction and display have been demonstrated in different biosensors. The following section summarizes influenza biosensors in the literature. Biosensors have been grouped based on the mechanism of bio-reception. Additionally, biosensors that have been studied in the context of poultry diagnosis specifically are also highlighted.

##### Glycan-based influenza virus biosensors

Glycans have been shown to function in the bioreceptor of biosensors for initial recognition of influenza virus. Glycans can be synthesized to mimic the carbohydrate structures present on human and chicken tracheal cells that influenza viruses bind when infecting a host. A rapid diagnostic assay that contained glycan-conjugated and anti-H5 antibody-conjugated nanoparticle probes was shown to recognize both H1 and H5 influenza proteins in solution (23). The assay utilized fluorescence energy resonance transfer (FRET) technology, where recognition of the (hemagglutinin) HA protein by the two probes brings them close together and because of special characteristics of the probes, fluorescence of the glycan-conjugated probe becomes quenched by the other. This allows for fluorescence to be measured and can indicate the presence of the protein and therefore the virus. This dual probe system of recognition has potential for incorporation into biosensor devices that do not require equipment to read fluorescence. Glycan based bio-reception has also been employed in other styles of sensors including: an impedimetric biosensor capable of detecting 13 H3N2 viral particles per microlitre of solution (24), a field effect transistor (FET) based biosensor that was capable of differentiating between H5 and H1 viruses by utilizing α-2,6 and α-2,3 sialic acids (25), glycan conjugated nanoparticle based sensors (26, 27), and a surface plasmon resonance (SPR) sensor (28). Additionally, using a series of glycan coated gold nanoparticles, Zheng et al. (27) were able to differentiate between 14 different influenza viruses using spectroscopy and colourimetric assays.

##### Antibody-based influenza virus biosensors

Antibodies have also been studied for use in the bioreceptor in rapid sensor systems for influenza virus (22). Using polyclonal antibodies from mice immunized with influenza M1 protein in an impedance electrochemical spectroscopy biosensor, Nidzworski et al. (29) were able to detect multiple strains of influenza. The M1 protein is similar among influenza A viruses, and therefore the researchers in the aforementioned study suggested that this biosensor is able to detect all influenza A viruses. Additionally, impedance biosensors have been developed with antibody recognition elements specific for peptides derived from the HA protein (30). Also, using two antibodies, including an anti-H5 antibody that sequestered influenza virus in solution, followed by an electrode mobilized anti-neuraminidase (N1) antibody, H5N1 virus was able to be more selectively detected in solution (31). Nevertheless, the sensor developed by Lum et al. (31) showed false positive reactions when tested with H5N2, but not H5N3, and this was likely due to shared structures between N1 and N2 that were both recognized by the anti-N1 antibody. Antibody coated nanoparticle based influenza sensory systems also have been studied, leading to development of a gold nanoparticle colourimetric sensor that recognized H3N2 (32) and a silver nanoparticle fluorescence based sensor that was capable of detecting H1N1 (33). Antibodies have also been used as recognition elements for H3N2 and H1N1 sensors using carbon nanotubes decorated with anti-influenza antibodies (34, 35). An immunogold biosensor that contained immobilized 2011 pandemic influenza virus specific anti-H1 antibodies and general anti-nucleoprotein antibodies has also been shown to be able to detect multiple strains of influenza A virus, while also differentiating detection against the pandemic strain (36). This technology could be important for the poultry industry if sensors can be designed that are specific for relevant and pathogenic strains of influenza virus.

##### Aptamer-based influenza virus biosensors

Another bioreceptor recognition element that has been employed for detection of influenza virus experimentally in rapid systems of detection are aptamers. Aptamers are short oligonucleotide or peptide sequences that form unique secondary structures allowing them to selectively bind to large or small molecules or macromolecules. Libraries of nucleotide sequences are generated and screened *in vitro* to identify sequences that recognize a target analyte with high specificity, and this technology has been employed to selectively detect different subtypes of influenza virus (37). They are favorable to glycans and antibodies due to their high level of affinity to their target, small size, stability, and reproducible synthesis. Multiple biosensors that employ aptamers for influenza virus recognition have been developed, and most have been specific for H5N1 detection (38). Additionally, aptamers covalently bound to gold nanoparticle electrodes have been shown to be able to capture H5N1 virus in H5N1 spiked human sera when utilized in a sandwich assay that additionally includes an H5N1 specific antibody (39). Aptamer coated nanoparticles have been demonstrated in rapid sensor devices as well. Pang et al. (40) used aptamer coated silver nanoparticles in a fluorescence assay to detect a recombinant H5 protein, and Fu et al. (41) used aptamer coated magnetic beads and glycan coated gold nanoparticles in an impedance biosensor to detect H5N1 virus. H5N1 specific SPR biosensors that employ aptamers as recognition elements have been developed using a single aptamer for detection (42), and using a sandwich aptamer model of detection, where a secondary aptamer conjugated to a silver nanoparticle was used to enhance the response of the SPR sensor (43). A sandwich aptamer fluorescence based biosensor has also been shown to detect H1N1 virus in solution (44). Very rapid impedance biosensors that utilize aptamers for detection have been developed against H5N1 (31) and H1N1 (45). In the above-mentioned studies, virus detection took only 15 and 30 min, respectively. Aptamers have also been reported as effective recognition elements in quartz crystal microbalance sensors for the detection of H5N1 virus. Of the three recognition elements mainly used for influenza virus detection, aptamers have shown to be very effective. Due to their enhanced specificity compared to antibodies and glycans, they are more capable of differentiating between different subtypes of influenza virus. The enhanced specificity also has led to the ability for detection of influenza virus at very low titers (31, 41, 44). A peptide aptamer based recognition element biosensor has also been developed for influenza virus detection (46). The ability to detect very low levels of influenza virus in complex biological samples is essential for an effective rapid biosensor device for poultry diagnostics.

##### Influenza virus biosensors for poultry diagnosis

Many of the technologies being developed for influenza biosensors are designed with the intention of human point-of-care diagnostics, however, these strategies can be applicable to poultry as well. AIVs replicate in the mucosal tissues of poultry species and for diagnostic purposes it is important that biosensors can accurately detect the virus from these sources. Additionally, some highly pathogenic avian influenza viruses gain entry to the systemic circulation of chickens and therefore detection from serum samples is also important. Of the multiple biosensors or rapid assays that have been developed for influenza virus detection, some have been tested using biological samples originating from poultry species. For example. using an aptamer recognition element in an impedance biosensor, the detection limit for an H5N1 virus spiked in chicken tracheal swab material was 1 HA unit/50 μl (HAU), while the limit for virus diluted in PBS was found to be 0.25 HAU (47). Impedance biosensors that utilize monoclonal antibodies against the H5 protein have demonstrated different results when compared to RT-PCR results from swabs from influenza virus infected chickens; Lin et al. (48) detected H5N1 in cloacal and oropharyngeal swabs with their impedance biosensor with equal sensitivity to RT-PCR, while Wang et al. (48) detected H5N2 in tracheal swabs with an equal level of sensitivity to RT-PCR, but at a lower sensitivity when cloacal swabs were the source. SPR biosensors have also been tested with chicken swab material. Wang et al. (37) used SPR to select a highly specific aptamer for H5N1 that demonstrated higher specificity than an anti-H5 monoclonal antibody when exposed to tracheal swabs spiked with other H5 influenza viruses, suggesting aptamers to be beneficial to monoclonal antibodies for use as a recognition element in biosensors for poultry diagnosis. Additionally, an SPR biosensor with an aptamer recognition element had a detection limit of 0.128 HAU for an H5N1 virus spiked in chicken throat swab samples (42), and a FET biosensor employing an anti-influenza nucleoprotein monoclonal antibody recognition element detected H9N2 virus from chicken cloacal swabs 4 days after experimental infection (18). Also, a quartz crystal microbalance biosensor with immobilized anti-H5 antibodies as a recognition element was shown to detect H5N1 spiked in chicken tracheal swabs with a detection limit of 0.128 HAU (49). Most biosensor devices applicable to poultry diagnostics have been developed for influenza virus, however there are other relevant pathogens that biosensor devices should be developed for. Accordingly, an SPR based biosensor with a bioreceptor that employed monoclonal antibodies against NDV has been reported (50). Future rapid detection biosensor studies should continue to focus on influenza virus detection from complex biological poultry samples, in addition to other pathogens that are relevant in the poultry industry. Also, detection from swabs of infected chickens as opposed to influenza spiked swabs should be a focus of future studies.

The ability to rapidly diagnose poultry disease and infection can be enhanced with biosensors and rapid detection assays. The specificity and speed that biosensor diagnostic technologies present is promising, however, these point-of-care devices require manual sampling. Biosensors would likely have to be used after clinical signs of disease are evident in poultry, where they will then replace lab-based methods of diagnosis, shaving days to weeks off the time to reach an official diagnosis. Nevertheless, the need for manual sampling is a detractor from biosensor-based diagnostics. Real-time sensing of infection in poultry is an ultimate target, as precious time can pass from when poultry initially become infected with a pathogen, to when clinical signs are noticed, and diagnostic devices can be used. Real-time detection of infection and disease in poultry is possible, and current strategies being researched include wearable sensors, and non-invasive methods of surveillance, such as a vocalization analysis and various imaging techniques. In addition to providing data to producers on animal health status in real-time, these detection methods are additionally beneficial as they decrease the need for routine human monitoring of poultry houses, thereby decreasing the chance for introduction of infectious agents into poultry flocks.

#### Technology for detection of poultry disease: wearable sensors

Wearable sensors for livestock management are already widely deployed by the agricultural industry, with multiple uses ranging from stress detection, behavior analysis, physiological monitoring, and detecting health and disease status of animals (16). Precision farming practices have made the usage of wearable sensors in other livestock sectors common, including dairy farming, where a variety of devices have been developed to track health and production variables of individual animals (51). Additionally, as sensory devices gain the ability to connect to the internet, producers can get a much better understanding of animal health and production about individual animals in real-time. A barrier to wearable sensors in the poultry industry is the number of birds that are managed on large poultry operations, as fitting every bird with sensory devices is not likely plausible. Nevertheless, fitting a proportion of the flock with sensors is possible, and the data generated from these birds can be used to assess total flock health (52).

Limited research has been done thus far on wearable sensors for pathogen detection in poultry. Wearable sensors that have been developed for poultry diagnostics have focused on H5N1 HPAIV. During infection with HPAIV, chickens and other poultry show severe clinical signs of disease that include lethargy and sometimes fever (53, 54). These physiological changes that occur during infection have been the target of detection of wearable sensors for influenza virus infection in poultry. Specifically, Okada et al. (55) first developed a wearable sensor that contained an accelerometer and thermistor probe that in total weighed 5.2 grams. The thermistor probe was able to assess chicken body temperature without making contact with the skin, and the accelerometer was used to assess activity level. The sensor was battery powered and to increase battery life to 2 weeks, the sensor functioned on and off on 20 s intervals. Okada et al. (55) tested the sensor by applying it to the abdominal skin of 4-week old chickens and challenging the chickens with three different strains of H5N1 HPAIV. In the study, all three strains of virus produced detectable decreases in activity prior to death, while two of three strains of virus led to increased body temperatures. From the data recorded in this experiment, an algorithm was designed to predict when chickens were infected. Three parameters, including a threshold body temperature exceeding 42°C or below 38°C, and a measure of activity that compared current activity level with those recorded over a period of 24 h prior were included in the calculation. Average time until death after infection was approximately 6–7 days depending on the strain of the virus, however, using the wearable sensor, detection of infection was noticed in chickens several hours before death, ranging from an average of approximately 6–36 h prior. This style of sensor has additionally been used to assess pathogenicity and transmission of high and low pathogenic influenza viruses in chickens (54, 56). Following the initial study by Okada et al. (55), a modified wearable sensor was then developed that focused solely on activity status by using an accelerometer and abolishing the thermistor probe (57). This was because data has demonstrated that not all strains of H5N1 induce a fever in infected chickens, and it is also found to be difficult to keep the thermistor probe close to the skin of the chicken. Okada et al. (57) demonstrated that using an accelerometer only biosensor, detection of infection prior to death occurred twice as fast when compared to the thermistor only biosensor.

Wearable sensors for detecting influenza virus infection typically focus on tracking specific physiological changes that occur during infection with an H5N1 HPAIV. As such, they provide an indication of infection several hours before death occurs, which is valuable to producers as an additional surveillance method combined with visual monitoring of birds. The main advantage of a wearable sensor, is that it can be constantly functioning and transmitting data remotely, and therefore can notify producers in near real-time as problems are detected. This saves even more time when compared to normal detection of a HPAIV infected flock, which would be likely only be noticed after morbidity and mortality has become widespread. Earlier detection allows for actions to be taken much sooner, including increasing biosecurity practices, culling of infected birds, or administration of prophylactic treatments. This not only can save producers from incurring higher losses, but it additionally can keep infection localized, decreasing the chance of an outbreak occurring. The downside to wearable sensors is a loss of specificity, as deceased levels of chicken activity or changes in body temperature could be induced by other factors (57). A possible solution to this could be utilizing wearable sensors for initial detection of decreased chicken health, followed by immediate use of a point-of-care biosensor assay that can specifically determine if influenza or some other poultry pathogen is the causative agent. Additionally, it remains to be seen if low pathogenic influenza virus infection in chickens would be detectable using wearable sensor technology. Low pathogenic influenza virus infection in chickens normally produces subclinical infection in poultry, although, depending on the strain of virus, some symptoms such as lethargy are suggested to occur in infection in some birds (53). Nevertheless, real-time surveillance of poultry for diagnostic purposes extends beyond wearable devices as other less invasive methods of disease detection exist.

#### Technology for detection of poultry disease: non-contact methods

Real-time analysis of poultry houses can be achieved using a variety of different surveillance methods. Techniques that can collect large and diverse data about poultry activity exist, but making use of the data that are collected is a challenge. Nevertheless, machine learning and other tools are making analysis of large complex data sets possible, and machine learning and big data analytics have been demonstrated in the analysis of data sets pertaining to livestock farming (58). The use of machine learning has already been employed to study a variety of wild and farmed animal behaviors (59). Conceivably, for the purpose of poultry disease diagnostics, machine learning algorithms could be employed to analyze poultry surveillance data and detect when disease behaviors or activities arise. Likely technologies that have potential for the poultry industry include vocalization and imaging analysis, in addition to the use of robotic surveillance.

##### Vocalization analysis

Poultry vocalization has been studied for a variety of different applications relevant to animal welfare, production, and health management. Vocalization analysis has been used to model and predict the weight and age of broiler chickens (60–62) and to detect the incidence of feather pecking in layer flocks (63). That said, to date there has not been a significant body of research created pertaining to infectious diseases of poultry and vocalization, especially for influenza virus. Sadeghi et al. (64) recorded broiler vocalization after infection with *Clostridium perfringens* and compared it to healthy broiler vocalization using Fisher's discriminate analysis to select five specific features that showed strong separation between healthy and infected birds. Then, using these five features a neural network was applied to detect healthy or infected chickens and was able to differentiate at an accuracy level of 66.6 and 100% on day 2 and day 8 post-infection, respectively. Additionally, by recording chickens infected with infectious bronchitis virus (IBV), and training a computer algorithm with manually labeled recordings, IBV infected chickens can be detected based on vocalization (65). The algorithm used in the aforementioned study was trained to recognize rales, which are commonly produced from IBV infected chickens, and was able to detect increased rale frequency days before clinical signs of disease were evident. Also using IBV infected chicken recordings, Rizwan et al. (66) compared an extreme learning machine algorithm and a support vector machine algorithm, and determined that both could detect increased frequencies of rales, but the support vector machine algorithm demonstrated decreased incidences of false positive results for rale detection. Vocalization analysis of poultry is promising for early detection of infectious disease and could be potentially used for high and low pathogenic AIV, as rales can be a sign of infection in chickens (53). An important note for future vocalization algorithms concerns the recordings that are used for training, as potential biases can be introduced if training data is skewed to favor other characteristics, such as chicken age or background noises.

##### Image analysis

Similar to vocalization, image analysis techniques can also be used to collect data on poultry at a low level of invasiveness and in real-time. Multiple image analysis strategies have been studied to monitor livestock, including image analysis, optical flow, and infrared imaging (67). Most poultry imaging studies have been focused on behavior, welfare, and production status of poultry (67, 68). Nevertheless, there are potential uses for diagnostics, as infection can lead to detectable differences in movement patterns and other attributes that can be detected with imaging technology. For example, using optical flow, which is a relatively simple imaging technique that measures changes in brightness in a series of images, infection with *Campylobacter* in chickens can be detected (69). Interestingly, as *Campylobacter* is a commensal bacterium in chickens, infection is thought to be subclinical, however subtle differences in chicken movements can be detected with optical flow patterns. This highlights the ability that machines and algorithms possess for detecting differences when compared to humans. Additionally, infrared thermal imaging can generate images demonstrating the superficial surface temperatures of poultry, and has been used to measure temperature changes associated with heat stress and diet (70). Infectious agents that induce fever in poultry could potentially be detectable with infrared thermal imaging due to changes in body temperature. However, research is needed to examine this further.

##### Robotic surveillance

Beyond the traditional surveillance mechanisms that entail digitally listening to and watching poultry, the use of robotics has also become a possible candidate for early detection of disease and infection. Multiple companies have begun producing robots that function in poultry houses to perform a variety of tasks. These robots are typically self-driving small vehicles that can enhance barn sanitation and increase chicken activity, in addition to performing other jobs (71). One task that these types of robots can perform is the detection of severely sick or dead chickens. Using a mounted camera to rapidly take multiple photos, robots can determine non-responsive and likely sick or dead poultry (72). These robots can provide early detection of disease, leading to removal of the animal and follow-up on the cause of death. Also, employing robots like the above-mentioned robots could decrease the need for humans to monitor poultry houses for sick birds, therefore, decreasing the chance of introduction of infectious agents into the poultry house.

Due to the ability of surveillance technologies and analytical tools like machine learning, complex data pertaining to large poultry flocks can provide valuable insights to the health and infection status of poultry. Detection of infection and disease in poultry is likely possible through the identification of subtle patterns of change of vocalization, activity, and physiology. These surveillance mechanisms provide potential for poultry to be tracked in real-time resulting in earlier detection of altered states of health. Moreover, point-of-care devices will provide the ability to quickly determine if infectious disease is specifically present. Taken together, early detection and rapid diagnostics will allow producers to respond to situations of infectious disease much sooner than previously possible. In the context of production in the poultry industry, this will decrease losses and inhibit the spread of infection to other birds. Decreasing transmission of infectious agents in poultry can also potentially diminish the threat of zoonotic transmission to humans, decreasing the threat of outbreaks associated with the future projected intensification of poultry production.

## Part II

### Exploiting data to predict disease emergence in poultry

The devastation that infectious disease can cause in the poultry industry, highlighted by AIV outbreaks such as the 2014/2015 North American outbreak, is a constant reminder that infectious disease must be contained quickly and efficiently in poultry. Early diagnosis of infectious disease in poultry is important for reducing losses due to infection, containing infection to a small group of birds, and decreasing the chance of zoonotic disease emergence in human populations. In addition, earlier detection of infectious disease could also become an important source of data for use in predictive models of emerging disease in poultry. Rather than reacting to situations of outbreak and disease, predicting when and where domestic poultry are at risk of becoming infected is ultimately desired. As intensification of poultry production increases, predicting disease emergence may be necessary to decrease potential losses attributed to infectious disease. Accurate predictive models require data associated with drivers of emerging disease, including environmental, geographical, farm, and internet-based data sources. Not only could predictive models incorporate dynamic sources of data to help with decision making, but doing so very quickly or potentially in real-time is becoming possible with the development and usage of on farm sensors in addition to development of internet of things (IoT) devices. The implementation of data-driven decision support systems is important as humans will likely struggle to make optimal decisions when faced with the dynamic data that will be available. With well-designed models, protective actions can be taken sooner in at-risk areas to prevent outbreaks, and these could include administering prophylactic treatments or increasing biosecurity measures. The following section describes two important data sources that have strong potential for inclusion in predictive infection models for poultry and a discussion of how big data and big data analytics will be essential in predictive systems of disease in poultry. Also, a summary of the challenges that are likely to be faced when implementing predictive models for poultry is included.

#### Data sources for predicting disease in poultry

##### Internet based data sources

The availability of data via the internet has great potential for industries or companies as it is produced constantly and is available for real-time analyses. Examples of data sources on the internet include search query and social media data from sources like Google and Twitter, respectively (14). Monitoring the incidence of human cases with various diseases has been demonstrated by tracking mentions of things such as flu-like symptoms on twitter or using data on the extent to which people search the internet on symptoms of flu. Such systems rely on people who become infected to use the internet to search about their condition, leading to measurable changes in the frequency of searches of certain keywords or phrases (73). For example, using Google Trends, which is a web-based tool for analyzing Google searches, incidence of regional influenza outbreaks can be detected prior to detection by the Centers for Disease Control and Prevention surveillance systems (74). Additionally, social media platform such as twitter also have been used to detect increased frequency of disease through user driven updates pertaining to their conditions. A Twitter based model has been demonstrated to predict the peak of seasonal influenza epidemics 6 weeks in advance with good accuracy (75). Despite the successes of detecting disease outbreaks in humans using web-based data, there are still challenges such as the lack of internet in some populations, in addition to biases generated by searches or posts that contain tracked words or phrases, but as a whole are not relevant to surveillance (73). Web-based data has allowed for syndromic surveillance and earlier warning of outbreaks in humans, yet for the purposes of poultry production this will not likely be able to be replicated entirely. Web-based systems that track human illnesses rely on users to generate data and this cannot be replicated for poultry, nonetheless, it has been shown that analyzing Twitter posts can efficiently summarize online reports to do with official AIV surveillance. Specifically, using four keywords related to poultry infection with AIV, Robertson and Yee (76) created an automated data extraction and analysis pipeline to analyze Twitter posts pertaining to AIV. In a period from 2015 to 2016, their model positively correlated AIV-related Twitter posts to avian influenza cases in birds reported by the World Organization for Animal Health (76). The use of Twitter therefore could act as a tool to provide access to various online reports pertaining to avian influenza surveillance and tracking these posts in real time could provide important data for monitoring and predicting the incidence of AIV in poultry. Nevertheless, it is important to take into account differences between internet based data generated by humans who become sick and internet based data related to outbreaks of poultry disease, as these differences will have implications in their respective efficacies for predictive capabilities.

##### Environmental data sources

Emergence of infectious diseases into both human and poultry populations are largely induced by interaction with the environment, specifically due to interaction with wildlife species that can transmit zoonotic infection. In humans, around 70% of zoonotic diseases originate from wildlife species and while some transmit directly to humans, many infect livestock species prior to human infection (77). The 2014 outbreak of H5 HPAIV in North America was discovered to have been brought from Japan and South Korea to northern Russia, and finally to North America via migratory birds infected with the virus (78). Phylogenetic analyses revealed further that infection in farmed poultry followed specific flyways that migratory birds use, including the pacific, central, and Mississippi flyways (78). Extensive surveillance in the USA prior to the outbreaks indicated statistically that HPAIV was not present in wild birds from 2006 to 2009 (79). This further demonstrates the role of migratory birds held in the 2015 outbreak and highlights the need for modified surveillance systems. An opportunity to enhance wild bird surveillance could be facilitated through better targeted surveillance strategies. Using network analyses of tagged migratory birds, more specific biological flyways of different species of birds in North America have been discovered, in addition to identification of important regions in North America for flyway determination (80). Incorporating this information into surveillance systems could lead to better aimed wild bird surveillance, possibly allowing for efforts to be focused into certain important regions. Additionally, wild bird surveillance for infectious pathogens relies on laboratory based methods that take days or weeks to determine results. The use of rapid detection biosensors and devices for surveillance of influenza virus or other relevant infectious pathogens could enhance the speed of detection for surveillance. By combining data from rapid diagnostic devices and data concerning specific flyway migration patterns into decision support systems, it could be possible to identify regions where farmed poultry are at increased risk of infection. For example, by incorporating multiple data sources including: past low pathogenic avian influenza virus infection in wild birds, geographical density of broiler farms, and distance to coastlines, Belkhiria et al. (81) developed a disease distribution map to predict HPAIV risk in the state of California. The map associated areas of California with the risk of incidence of HPAIV infection, which would help to direct appropriate actions in the case of an outbreak. Predictive risk maps could also be designed to incorporate additional data sources, such as farm generated diagnostic data. In addition, risk maps or predictive models should be able to incorporate data in real-time in order to provide accurate predictions based on current events.

#### Big data analytics and decision support systems

To best predict when and where farmed poultry are at risk of infection, predictive models must integrate multiple sources of data into decision support systems. These include multiple web-based data sources, data from environmental inputs highlighted by, but not limited to, migratory bird surveillance, poultry farm location data, and importantly should include data collected by diagnostic devices and sensors present on poultry farms. Incorporating data from multiple farms that each contain multiple devices will produce enormous volumes of data, especially considering that poultry surveillance data could include media such as images or recordings taken from the barn to assess infection status. Similarly, data collected from these various sources present a very heterogenous pool of data for a predictive model to incorporate. Importantly, the best predictive model of emerging disease in poultry should be able to incorporate data and function in real-time, for rapid identification of areas that are at risk. Incorporating dynamic data into decision support systems is a problem fit for big data analytics. Big data can be described by datasets that contain the “v's,” including the most applicable attributes: volume, variety, and velocity (82). Big data analytics can add value to industries or corporations in many ways (83). For the poultry industry, it presents a way to incorporate voluminous and heterogenous pools of data into decision support systems that can function in real-time to provide an indication of where disease may emerge in farmed poultry, allowing for actions to be taken in a specific directed manner.

#### Challenges and barriers to implementing predictive infection models for poultry

Developing a harmonized system to predict disease emergence in poultry will be faced with multiple challenges ranging from problems to do with infrastructure to issues of data governance. Sensors and biosensors for diagnosis of poultry infection are still largely in development and will require more research to produce technology that can function accurately in a commercial poultry house setting. Another barrier is the lack of internet access on farms, which are often located in rural settings. Even in Canada, a significant portion of farms do not have access or consistent access to broadband (84). A lack of broadband inhibits implementation of new technologies and devices that require internet connection to function and provide data in real-time, such as wearable sensors for poultry. For predictive models to function in real-time or close to real-time, data collection should also be automated and collected continuously. This is feasible for certain devices, such as wearable sensors, and barn imaging/recording devices, although it is not likely feasible at this time for specific biosensors or for surveillance data from environmental sources. When considering farm technologies, there will be a need for producers to obtain education and skills related to the new technologies that are implemented. Another challenge predictive models face will be dealing with bias and noise associated with certain data sources. For example, web-based sources are known to generate lots of meaningless data and noise, which must be removed from analyses (73). A major issue going forward when considering harmonized predictive models involving multiple stakeholders and farmers pertains to data governance, which will likely be a concern of farmers (85). For the most accurate prediction of emerging disease, models should incorporate data from as many farms as possible, however producers are likely to not want to make their data publicly available. Anonymization of data is possible, but it has been demonstrated that techniques of data reidentification are becoming simple to perform (86). Amid the value that predictive models of emerging disease could provide for the poultry industry, multiple challenges are still faced, and should be a source of innovation and initiative going forward.

## Conclusions

As poultry production increases, poultry farms will be forced to become larger in size with greater numbers of birds. The demand for increased production will force producers to be efficient, and decreasing losses will be crucial, yet highly populated poultry farms will likely only increase the chance of incurring losses due to infectious disease. Simply put, traditional systems of monitoring disease and infection will not be sufficient if future production goals are to be achieved. Instead, rapid detection systems that constantly monitor poultry for disease can complement pre-existing systems of infectious disease detection and diagnosis. Rapid real-time detection can alert and locate producers to problems immediately. Additionally, biosensors will provide producers with a specific diagnosis that is performed on-site. The combination of early detection and rapid diagnosis provides great value to producers as it allows for immediate action to be taken in order to prevent any subsequent spread of infection to other birds, therefore saving potential losses that likely would have occurred had traditional methods been used. As these devices become common on farms, they will also provide data that could help to predict emerging diseases in poultry. In addition to farm generated data, web-based, environmental, and geographical data will also become important for collection and inclusion in predictive models. The dynamic data that will be included in these models will require systems of big data analytics in order to account for the volume and variety of the data in addition to the need for real-time analysis. The implementation of technologies in the poultry production industry that enhance detection, diagnosis, and prediction of infectious diseases currently faces multiple challenges, however they will be necessary to achieve rates of production required in the future.

## Author contributions

JA, EF, RD, and SS produced an outline and performed the literature review. JA wrote the manuscript. EF, RD, and SS critically edited the manuscript. All authors read and approved the manuscript.

### Conflict of interest statement

The authors declare that the research was conducted in the absence of any commercial or financial relationships that could be construed as a potential conflict of interest.
