# Corrigendum: Detecting and Predicting Emerging Disease in Poultry With the Implementation of New Technologies and Big Data: A Focus on Avian Influenza Virus

**DOI:** 10.3389/fvets.2018.00337

**Published:** 2019-01-22

**Authors:** Jake Astill, Rozita A. Dara, Evan D. G. Fraser, Shayan Sharif

**Affiliations:** ^1^Department of Pathobiology, University of Guelph, Guelph, ON, Canada; ^2^School of Computer Science, University of Guelph, Guelph, ON, Canada; ^3^Arrell Food Institute and Department of Geography, Environment and Geomatics, University of Guelph, Guelph, ON, Canada

**Keywords:** influenza virus, poultry, rapid diagnosis, big data, biosensor, infectious disease

Two author names were incorrectly provided as “Rozita Dara and Evan Fraser.” The names should be “Rozita A. Dara and Evan D. G. Fraser.” The order of the authors has also been corrected, as this was mistakenly changed during the manuscript revisions. Rozita A. Dara should appear as the second author and Evan D. G. Fraser should appear as the third author.

Additionally, there was an error in affiliation 3. Instead of the “Department of Geography, Environment and Geomatics, University of Guelph, Guelph, ON, Canada,” it should be the “Arrell Food Institute and Department of Geography, Environment and Geomatics, University of Guelph, Guelph, ON, Canada.”

Lastly, there was an error in the keywords listed. A correction has been made to the Keywords.

“Keywords: influenza virus, poultry, rapid diagnosis, big data, biosensor, infectious disease”

The authors apologize for these errors and state that they do not change the scientific conclusions of the article in any way. The original article has been updated.

